# Semi-supervised contrastive learning variational autoencoder Integrating single-cell multimodal mosaic datasets

**DOI:** 10.1186/s12859-025-06239-5

**Published:** 2025-08-04

**Authors:** Zihao Wang, Zeyu Wu, Minghua Deng

**Affiliations:** 1 Biomedical Interdisciplinary Research Center, Peking University, Yiheyuan Road, Beijing, 100871 China; 2 School of Mathematical Sciences, Peking University, Yiheyuan Road, Beijing, 100871 China; 3 Center for Statistical Science, Peking University, Yiheyuan Road, Beijing, 100871 China; 4 Center for Quantitative Biology, Peking University, Yiheyuan Road, Beijing, 100871 China

**Keywords:** Mosaic intergrate, Single-cell multimodal, Batch effect

## Abstract

As single-cell sequencing technology became widely used, scientists found that single-modality data alone could not fully meet the research needs of complex biological systems. To address this issue, researchers began simultaneously collect multi-modal single-cell omics data. But different sequencing technologies often result in datasets where one or more data modalities are missing. Therefore, mosaic datasets are more common when we analyze. However, the high dimensionality and sparsity of the data increase the difficulty, and the presence of batch effects poses an additional challenge. To address these challenges, we proposes a flexible integration framework based on Variational Autoencoder called scGCM. The main task of scGCM is to integrate single-cell multimodal mosaic data and eliminate batch effects. This method was conducted on multiple datasets, encompassing different modalities of single-cell data. The results demonstrate that, compared to state-of-the-art multimodal data integration methods, scGCM offers significant advantages in clustering accuracy and data consistency. The source code of scGCM can be accessed at https://github.com/closmouz/scCGM.

## Introduction

Currently, single-cell multimodal data integration has become an important research direction in bioinformatics, allowing for a more comprehensive understanding of biological systems through integrated analysis. Its goal is to integrate and analyze molecular information across different levels at the single-cell level to obtain more comprehensive and detailed information on cell function and state for subsequent downstream analysis. Although experimental technologies capable of measuring multiple modalities of data from the same cell population are continually advancing, different sequencing technologies often result in the loss of one or more modalities in a single data sets. Therefore, mosaic data sets are more common in single-cell data analysis, and we need to consider integrating these data to obtain a richer, low-dimensional embedding of cellular biological information ([1]).

The rapid development of single-cell multimodal sequencing technologies has made it possible to simultaneously obtain genomic, transcriptomic, epigenomic, and other types of data at the single-cell level. However, there are still some issues that need to be addressed. One is data sparsity. The amount and quality of data vary significantly across different modalities. The high-dimensional characteristics of single-cell data lead to high data sparsity. Ensuring data reliability is a critical issue. And other one is batch effects. Data from different modalities originate from different experimental platforms and sequencing technologies, even the same modality data can produce batch effects and technical noise in different experimental environments. Correcting batch effects to ensure data accuracy is a major challenge.

However, this has also introduced new challenges in how to effectively integrate and analyze these multimodal data. Compared to some conventional methods, such as Seurat ([2, 3]), which uses canonical correlation analysis (CCA) in scRNA transcriptome analysis, Liger ([4]), which uses non-negative matrix factorization, and harmony ([5]), which performs dimensionality reduction with principal component analysis (PCA) followed by soft k-means clustering, all of these reduce the dimensionality of each modality’s data into the same embedded space. The emergence of weighted nearest neighbor (WNN) has allowed for more effective integration of different modality data in the embedded space, computing weights for each modality’s data and combining them, greatly improving the integration effect of different modality data in the embedded space ([2]). However, they only learn our single-cell data through linear network, which fails to capture the nonlinear structures in the single-cell data.

Recently, some tools specifically designed for mosaic data integration have emerged. For example, scMDC ([6]) uses autoencoders to build deep learning clustering models; Cobolt ([7]), totalVI ([8]), and MultiVI ([9]) use Variational AutoEncoders (VAE) ([10]) for dimensionality reduction and integration of modalities, while Unipoert ([11]) and SCOT ([12]) use optimal transport for integration. However, most tools are only suitable for bimodal single-cell datasets, with fewer methods available for integrating trimodal sequencing data. Frameworks proposed by MOFA+ ([13]) and mutilta ([14]) can only integrate complete trimodal data, while GLUE ([15]) is designed for mosaic data integration but requires some biological knowledge, such as the relationships between genes, surface proteins, and chromatin accessibility regions, to construct an integration map. Stabmap ([16]) integrates by embedding all modalities of cells into the same space and following the shortest topological path in the original mosaic data space. Multigrate ([17]) integrates data by obtaining approximate distributions from the data obtained by modular encoders for each modality and can also complete missing modalities. scMHNN ([18]) uses hypergraphs for data integration, combining multimodal nearest neighbor graphs with weights to learn topological relationships. Mowgli ([19]) decomposes multimodal data using non-negative matrix factorization and then uses optimal transport (OT) for modality alignment, learning a shared latent representation. SnapATAC2 ([20]) uses spectral clustering to map high-dimensional data into latent space, significantly reducing computational efficiency and cost, and integrates by learning weights and stitching different modalities of single-cell data together.

Although existing methods have made some progress in multimodal data integration, they still face several key challenges. Batch effect correction is a major issue in the process of multimodal data integration. Since the cell populations and sequencing methods differ in each experiment, the resulting datasets often exhibit significant batch effects. This effect can significantly impact the quality of data integration, making it difficult to directly compare data from different batches. In existing data integration methods, cell type information is often overlooked. However, cell type information is crucial for downstream analyses, such as single-cell clustering. Accurate cell type annotation can significantly enhance the biological relevance of the analysis results.

To address these challenges, this paper proposes a modular integration framework based on Variational Autoencoder called scGCM(single-cell Graph Contrastive Modular variational autoencoder). The main task of scGCM is to integrate single-cell multimodal mosaic data and eliminate batch effects. This framework represents single-cell data as graph structures and utilizes graph structures to preserve both local and global features of cells, maintaining the topological structure of the data during dimensionality reduction ([21]). Additionally, the framework employs neighborhood graphs and contrastive learning ([22–24]) to effectively eliminate batch effects, ensuring robust integration of different modalities within the embedded space. This approach not only enhances the accuracy of data integration but also improves the identification of cell types. This method was conducted on multiple datasets, encompassing different modalities of single-cell data, the results demonstrate that, compared to state-of-the-art multimodal data integration methods, scGCM offers significant advantages in clustering accuracy and data consistency. The comparison of these results highlights the potential of the scGCM approach for multimodal data integration and provides methodological support for future single-cell research (Fig. 1).

## Method

**Fig. 1:**
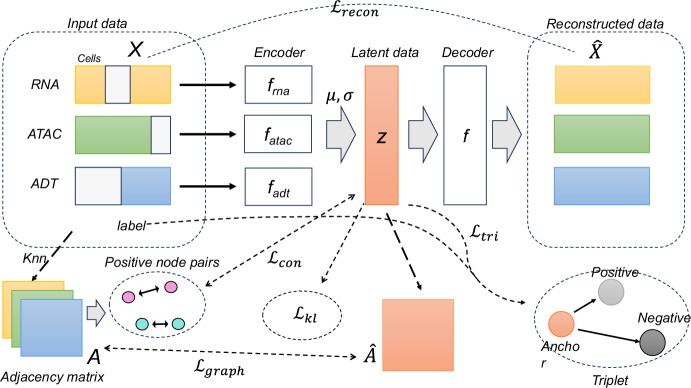
Overview of the scGCM framework. This figure illustrates the entire model structure. A modular variational autoencoder integrates single-cell multimodal mosaic data, learning their latent representations, and then decodes the learned representations through a neural network to reconstruct the data. The difference between the reconstructed data and the input data, along with the KL divergence of the latent representations, helps train the VAE model framework. Additionally, the model’s performance is enhanced by preserving consistency between the adjacency matrix of the input data and the adjacency matrix of the latent representations, and by applying contrastive learning to the positive sample pairs in the adjacency matrix. Finally, triplets are constructed using the labels of the input data to train the latent representations, improving the model’s ability to distinguish data types

### Modular variational autoencoder

For multimodal data, let’s assume there are *M* modalities in total. If we consider each subset $m\subset \{1,2,\ldots,M\}$(where *m* represents the included modalities), separately for neural network training, then the total number of data categories we need to handle would be as high as ${2}^M-1$. In this case, we would need to train a total of ${2}^M-1$ neural networks, which would be extremely cumbersome. However, if we can learn an encoder for each modality separately and then integrate them later, we would only need to train *M* neural networks. To achieve this, we introduce the Modular Variational Autoencoder. Let $x_n$ correspond to the latent representation in the embedded space as $z_n$. For each single cell *n*, we decompose the joint distribution of all its variables in the latent space:

1 $$\begin{aligned} p(x_n,z_n)=p(z_n)p(x_n|z_n) \end{aligned}$$

We assume that $z_n$ follows a Gaussian distribution, i.e., $p\left( z\right) =Normal\left( z|0,I\right)$ is the prior Gaussian distribution of the latent representation. The mean $\mu$ and variance $\sigma$ of the posterior Gaussian distribution in the latent space are learned through the neural network encoder, which can be achieved by maximizing the Evidence Lower Bound (ELBO) using a stochastic gradient variational Bayes algorithm. For a single cell data $x_n$, its Evidence Lower Bound (ELBO) can be computed as follows:

2 $$\begin{aligned} \begin{aligned} ELBO(\theta,\phi,x_n)=&\mathbb {E}_{q_\phi }\left( z| x_n\right) log\frac{p_\theta \left( x_n,z\right) }{q_\phi \left( z| x_n\right) }\\ =&\mathbb {E}_{q_\phi }\left( z| x_n\right) \left( \sum _{m\in \mathcal {M}_{n}}{logp_\theta \left( x_n^m| z\right) }\right) \\&-KL\left( q_\phi \left( z| x_n\right)| p\left( z\right) \right) \end{aligned} \end{aligned}$$

where $\phi$ represents the parameters of the encoder model learned by the neural network, and $\theta$ represents the parameters of the decoder model, denoted as $\theta =\{\theta ^m\}_{m\in \mathcal {M}}$. The first part is the expected log-likelihood estimate, which is essentially similar to a reconstruction loss (i.e., comparing the differences between the new data and the original data), encouraging the newly generated data using *z* to be close to the original data. The second part measures the difference between the prior distribution and the variational approximation distribution. Here, we also assume that the different modalities are independent Gaussian distributions under the latent representation. $q_\phi \left( z| x_n\right)$ is the variational approximation learned through the encoder, used to estimate our posterior distribution $p\left( z| x_n\right)$. Additionally, $KL=\left( \cdot|\cdot \right)$ denotes the KL divergence between two distributions. Inspired by Multigrate, we use a Product of Experts approach to achieve variational inference, further decomposing $p\left( z| x_n\right)$. As a result, we can approximate the final posterior distribution in the latent space by combining all modalities:

3 $$\begin{aligned} \begin{aligned} P\left( z| x_n\right) =&\frac{1}{\prod _{m\in {\mathcal {M}}_{n}} p\left( x_n^m\right) }p\left( z,x_n\right) \\ =&\frac{1}{\prod _{m\in {\mathcal {M}}_{n}} p\left( x_n^m\right) }\left( p\left( x_n| z\right) p\left( z\right) \right) \\ =&\frac{1}{\prod _{m\in {\mathcal {M}}_{n}} p\left( x_n^m\right) }\prod _{m\in {\mathcal {M}}_{n}}{\frac{p\left( z| x_n^m\right) p\left( x_n^m\right) }{p\left( z\right) }p\left( z\right) }\\ \approx&\frac{1}{\prod _{m\in {\mathcal {M}}_{n}} p\left( x_n^m\right) }\prod _{m\in {\mathcal {M}}_{n}}{\frac{q_\phi \left( z| x_n^m\right) p\left( x_n^m\right) }{p\left( z\right) }p\left( z\right) }\\=&\prod _{m\in {\mathcal {M}}_{n}}{\frac{q_\phi \left( z| x_n^m\right) }{p\left( z\right) }p\left( z\right) } \end{aligned} \end{aligned}$$

Let $q_\phi \left( z| x_n^m\right)$ be the variational approximation of the true distribution $p\left( z| x_n^m\right)$. For the convenience of neural network learning, we denote $\widetilde{q_\phi }\left( z|x_n^m\right) =\frac{1}{C_m}\frac{q_\phi \left( z| x_n^m\right) }{p\left( z\right) }$, where $C_m$ is the normalization coefficient of the distribution function. Thus, we can calculate:

4 $$\begin{aligned} \widetilde{q_\phi }\left( z| x_n\right) \propto p\left( z\right) \prod _{m\in \mathcal {M}_{n}}{\widetilde{q_\phi }\left( z| x_n^m\right) } \end{aligned}$$

We usually assume that $q_\phi \left( z| x_n^m\right)$ follows a diagonal Gaussian distribution, and in this case, the variational approximation distribution $\widetilde{q_\phi }\left( z| x_n^m\right)$ would also be a diagonal Gaussian distribution. In this case, we consider the encoder learning the mean $\mu _n^m$ and variance $\sigma _n^m$ of the latent representation *z* under the condition $x_n^m$, and we have:

5 $$\begin{aligned} \widetilde{q_\phi }\left( z| x_n^m\right) =Normal\left( z|\mu _n^m,diag\left( \sigma _n^m\right) \right) \ for \ m\in \mathcal {M}_{n} \end{aligned}$$

where, $diag\left( \cdot \right)$ denotes converting a vector into a diagonal matrix. From equations (4) and (5), we can see that the variational approximation we need is the product of a series of Gaussian probability distributions. Here, we can calculate the mean and variance of the Gaussian distribution that *z* follows under the condition $x_n^m$:

6 $$\begin{aligned} \mu _n=\left( \sum _{m\in \mathcal {M}_{n}}\frac{\mu _n^m}{\nu _n^m}\right) \circ \nu _n \end{aligned}$$

7 $$\begin{aligned} \nu _n=\left( 1+\sum _{m\in \mathcal {M}_{n}}\frac{1}{\nu _n^m}\right) ^{-1} \end{aligned}$$

where, $\circ$ represents the Hadamard product. The above equation is derived directly through probability distribution calculations, so it will not be elaborated on further here.

Next, we generate the latent representation $z_n$ of cell $x_n$ using the probability distribution of *z* learned here. Subsequently, we reconstruct $\widehat{x_n}$ for each modality using their respective decoders. Here, unlike the conventional VAE model, which uses the negative log-likelihood to represent the reconstruction loss, we use the more intuitive mean squared error (MSE) to represent the loss:

8 $$\begin{aligned} \mathcal {L}_{recon}=\sum _{n=1}^{N}\left| \left| x_n-\widehat{x_n}\right| \right| ^2 \end{aligned}$$

Then, the previous KL divergence is denoted as:

9 $$\begin{aligned} \mathcal {L}_{kl}=KL\left( q_\phi \left( z| x_n\right)| p\left( z\right) \right) \end{aligned}$$

### Topological structure relationships

We generally believe that data from similar cells should be similar across various modalities, such as gene expression, meaning that their distances in the topological space should be relatively small, while different types of cells should be far apart from each other. Therefore, we consider using the nearest neighbor graph of the raw data to preserve the original topological relationships for integration. Additionally, we believe that for similar cells, even across different batches, their spatial distances relative to other cells should remain relatively close. We use the topological relationships between cells to integrate multimodal mosaic data and eliminate batch effects.

First, we need to construct the corresponding nearest neighbor graph, and we use cosine distance to represent the similarity between cells:

10 $$\begin{aligned} sim\left( x_i,x_j\right) =\frac{x_i\cdot x_j}{\left| x_i\right| \left| x_j\right| } \end{aligned}$$

We use the calculated distances to construct the K-nearest neighbor graph, denoted as $G=\left( A,X\right)$, where *X* represents the cell count matrix, and $A\in \mathbb {R}^{N\times N}$ represents their adjacency matrix. If single cells *i* and *j* are mutual neighbors, then $A_{ij}=A_{ji}=1$, and all others are 0. In practice, the significant features and properties of each modality are different, and their neighbor relationships are closely linked to their modality, so their neighbor graphs may vary. Here, the model calculates the adjacency matrices $A^{rna}$, $A^{atac}$, and $A^{adt}$ for each modality. Based on the concept of Graph Convolutional Networks, we believe that even in the latent space, our latent representation *Z* should still preserve this topological relationship. We aim for the adjacency matrix $A^r$ obtained from *Z* to be as similar as possible to the adjacency matrix obtained from the original data, so we can incorporate this into the loss function. We use the graph cross-entropy:

11 $$\begin{aligned} A^r=sigmoid\left( Z^TZ\right) \end{aligned}$$

12 $$\begin{aligned} \mathcal {L}_{graph}= -\frac{1}{N}\sum _{m \in \mathcal {M}_{n}}\sum _{i,j}A_{ij}^{m}logA_{ij}^r \end{aligned}$$

where, $sigmoid\left( \cdot \right)$ is the sigmoid function. By maximizing the distances between neighboring points in the embedded space (minimizing the above loss function), we can preserve the topological relationships between cells, thereby making the learned latent representations more discriminative and reducing batch effects by aligning cells with similar biological states across different batches.

### Contrastive learning enhance

To enhance the heterogeneity between the original cell data, we introduce contrastive loss for learning. Our goal is to bring similar cells closer together in the multimodal data sets while pushing dissimilar cells as far apart as possible. Here, the model uses the original adjacency matrix obtained in the previous section to find our positive sample pairs. For $A^{rna}$, $A^{atac}$, and $A^{adt}$, we randomly select *T* pairs of neighboring cells as our positive sample pairs, denoted as $S^{rna}=\{\left( z_{\alpha _i},z_{\alpha _i^{pos}}\right) \}_{i=1,2,\ldots,T}$, $S^{atac}$ and $S^{adt}$, where *T* is the number of sample pairs. We calculate the infoNCE loss for each positive sample pair:

13 $$\begin{aligned} \ell (z_{\alpha _i},z_{\alpha _i^{pos}})=log\frac{e^{\left( \frac{sim(z_{\alpha _i},z_{\alpha _i^{pos}})}{\tau }\right) }}{\sum \limits _{j=1}^{T}\mathbb {I}_{j\ne i}e^{\left( \frac{sim(z_{\alpha _i},z_{\alpha _j^{pos}})}{\tau }\right) } +\sum \limits _{j=1}^{T}e^{\left( \frac{sim(z_{\alpha _j},z_{\alpha _i^{pos}})}{\tau }\right) }} \end{aligned}$$

14 $$\begin{aligned} \mathcal {L}_{con}^{m}=-\frac{1}{2T}\sum _{i=1}^{T} \left( \ell \left( z_{\alpha _i},z_{\alpha _i^{pos}}\right) +\ell \left( z_{\alpha _i^{pos}},z_{\alpha _i}\right) \right) \end{aligned}$$

15 $$\begin{aligned} \mathcal {L}_{con}=\sum _{m \in \mathcal {M}_{n}}\mathcal {L}_{con}^{m} \end{aligned}$$

The contrastive loss for each modality is calculated based on its own positive sample pairs, where $sim\left( \cdot \right)$ denotes cosine similarity, $\mathbb {I}_{j\ne i}$ is an indicator function that equals 1 when $i\ne j$, and $\tau$ is a temperature hyperparameter. Here, we only consider the positive sample pairs, while all other data points are treated as negative samples relative to these two positive samples. By minimizing the above contrastive learning loss, we can pull the positive sample pairs closer together and push the negative samples further apart, thereby learning more accurate latent representations of the cells.

### Triplet semi-supervised learning

We have some data with cell type, and to fully utilize this known information, we construct a sample triplet based on this dataset, for example, (sample point, positive sample point, negative sample point), denoted as $S^{tri}=\{\left( a_i,p_i,n_i\right) \}_{i=1,2,\ldots,H}$, where $a_i$ is a randomly selected sample point, $p_i$ is a randomly selected sample point with the same cell type, and $n_i$ is a randomly selected sample point with a different cell type. *H* is the number of triplets. Here, we define the triplet loss:

16 $$\begin{aligned} \ell \left( a,p,n\right) =max\left( d\left( a,p\right) -d\left( a,n\right) +m,0\right) \end{aligned}$$

Here, *d* represents the distance metric, and we use Euclidean distance. *m* represents the margin, which is the maximum acceptable difference between the distances of the positive and negative samples relative to the anchor point. Here, our total loss is:

17 $$\begin{aligned} \mathcal {L}_{tri}=-\frac{1}{H}\left( \sum _{i=1}^{H}\ell \left( a_i,p_i,n_i\right) \right) \end{aligned}$$

We optimize our triplet loss by minimizing the distance between positive sample pairs and maximizing the distance between negative sample pairs. This is similar to the idea of contrastive learning, where we bring cells of the same class closer together and push cells of different classes further apart. By fully utilizing information from the cell type, this approach can further enhance the integration of our multimodal datasets and reduce batch effects by aligning cells of the same type across different batches.

### Model training

To train the model’s encoder and decoder, taking into account all the equations (8, 9, 12, 15, 17), we define our total loss function:

18 $$\begin{aligned} \mathcal {L}=\lambda _1\mathcal {L}_{recon}+\lambda _2\mathcal {L}_{KL} +\lambda _3\mathcal {L}_{graph}+\lambda _4\mathcal {L}_{con}+\lambda _5\mathcal {L}_{tri} \end{aligned}$$

where, $\lambda _1$, $\lambda _2$, $\lambda _3$, $\lambda _4$, $\lambda _5$ are the weight coefficients, representing the importance of each loss in the overall model training.

## Results

### scCGM integration of transcriptomics and chromatin accessibility paired data

**Fig. 2:**
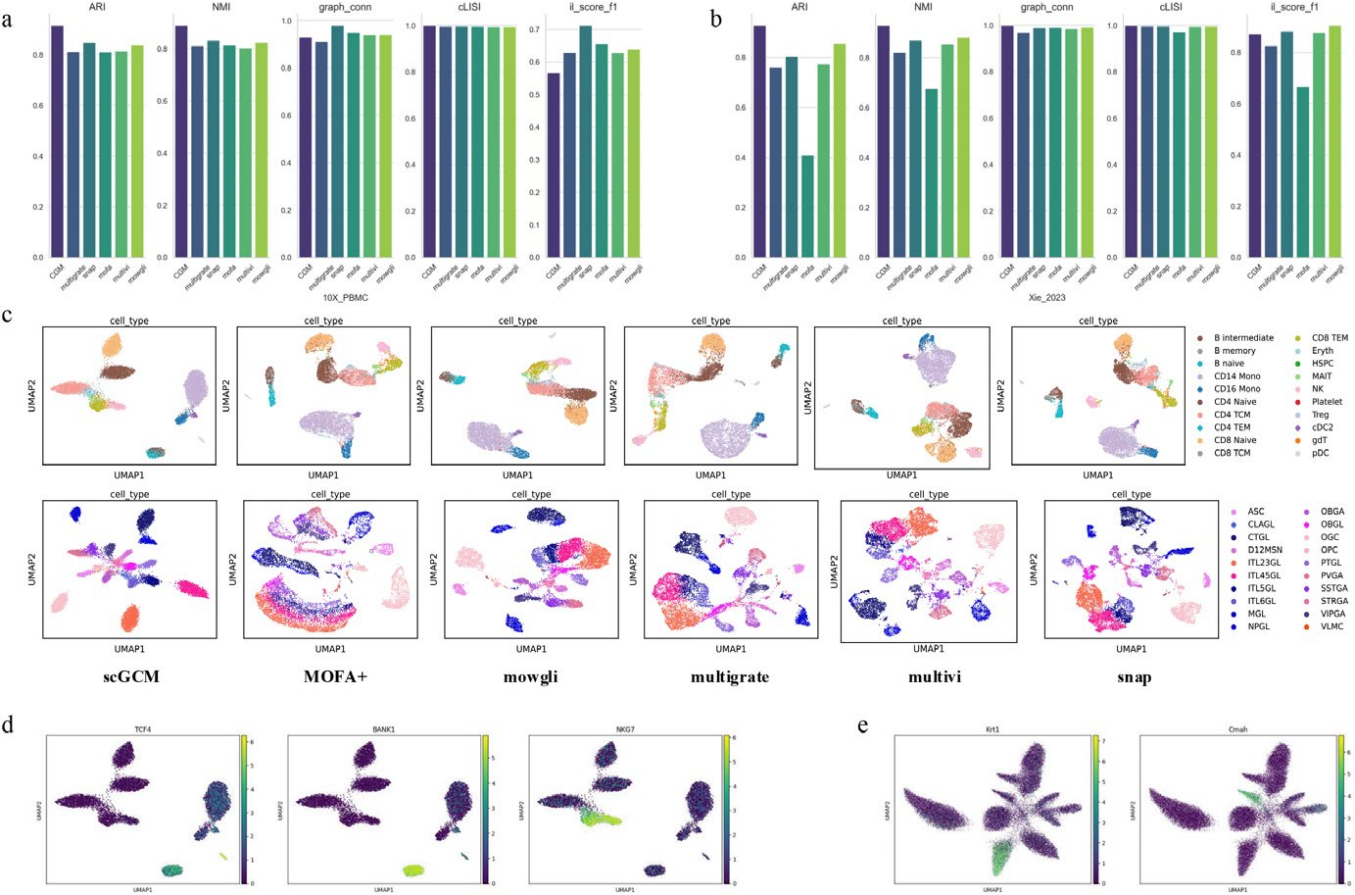
Evaluation obtained with scGCM on RNA and ATAC paired data. **a** benchmarking of performance in $10X\_PBMC$ dataset. **b** benchmarking of performance in $Xie\_2023$ dataset. **c** UMAP visualization of cell embeddings obtained by scGCM and five other strategies in $10X\_PBMC$ and $Xie\_2023$ dataset. **d** Gene expression of TCF4, BANK1 and NKG7 over all single-cell samples in $10X\_PBMC$ dataset. **e** Gene expression of Krt1 and Cmah over all single-cell samples in $MA\_2020$ dataset

We benchmarked the model framework on four RNA and ATAC paired datasets to verify its effectiveness in integrating single-cell multimodal paired data. These datasets include the $10X\_PBMC$ dataset of human peripheral blood mononuclear cells, the $Ma\_2020$ dataset of mouse epidermal cells, the $Chen\_2019$ dataset of the mouse cerebral cortex, and the $Xie\_2023$ dataset of the mouse frontal cortex. We used the Leiden algorithm based on KNN nearest neighbor graphs for clustering and visualized the integrated results using Uniform Manifold Approximation and Projection (UMAP) ([25]).

In Fig. 2a–b, we evaluated the performance of various methods, including Normalized Mutual Information (NMI), Adjusted Rand Index (ARI), Graph Connectivity, Graph Integrated LISI (Graph iLISI), and Graph Cell Type LISI (Graph cLISI). These metrics are displayed in bar charts showing the performance of all methods. In Fig. 2c, we present the UMAP visualization of the latent representations learned by these methods. In Fig. 2d–e, we present the UMAP visualization of gene expression over all single-cell samples.

From the results, it can be seen that for the $10X\_PBMC$ dataset, scGCM achieved the highest NMI and ARI, reaching about 0.9, while the performance of other methods was relatively close, around 0.8. scGCM performed similarly to Multigrate, Snap, MOFA+, MultiVI, and Mowgli in terms of Graph Connectivity and cLISI. However, Snap achieved the highest $il\_score\_f1$ in identifying rare label types, exceeding 0.7, while scGCM showed weaker performance in recognizing these rare cell types. Nevertheless, from the UMAP plot, scGCM was able to clearly distinguish cell types with larger populations. Based on previous studies, TCF4 is a marker gene for pDC cells, while the BANK1 gene plays a critical role in B cell signaling, activation, and differentiation. It regulates the B cell receptor (BCR) signaling pathway, ensuring that B cells effectively respond to external stimuli and maintain immune system balance and function. Additionally, the NKG7 gene is highly expressed in natural killer (NK) cells and CD8+ effector memory T cells (CD8 TEM), contributing to cytotoxic responses and aiding these cells in combating viral infections and tumor cells. Here we showed the UMAP visualization of cell markers, we can observe the consistency of ground-truth cell type with corresponding well-known cell markers. This further consolidates the intergration ability of scGCM.

For the $Ma\_2020$ dataset (Figure S2), scGCM’s NMI and ARI were significantly better than all other methods, reaching 0.8. The Snap method, based on spectral clustering, performed slightly better than the other methods by about 0.1. Graph Connectivity and cLISI results were generally consistent across all methods, with Snap achieving the highest $il\_score\_f1$ of 0.8. As seen in the UMAP plot (Figure S4), other methods had blurred cell type identification, while scGCM successfully clustered different cell types distinctly. Based on the UMAP visualization of marker genes and existing knowledge (Figure S6), we know that the Krt1 gene serves as an important marker in the process of skin cell differentiation. As keratinocytes differentiate from the basal layer to the spinous layer, the expression of Krt1 gene gradually increases. Although there is limited research on the Cmah gene in ORS cells, existing knowledge suggests that, since the Cmah gene is inactive in humans, it may contribute to the aging or degeneration of hair follicles, providing a direction for future research.

In the $Chen\_2019$ dataset (Figure S1), scGCM and Snap had similar NMI and ARI performance, significantly outperforming other methods, reaching 0.75$-$0.8. The Mowgli method, based on non-negative matrix factorization, performed the worst, with values of only 0.2 and 0.4. Graph Connectivity and cLISI were mostly even across methods, with Snap leading in $il\_score\_f1$, while scGCM ranked third, achieving over 0.5. From the UMAP plot (Figure S3), only scGCM and Snap clearly separated cell types, while the results of other methods were more blurred.

### scCGM integration of transcriptomics and protein data

We conducted benchmarking on a set of RNA and ADT paired datasets, comparing our developed model framework with current state-of-the-art multimodal paired data integration methods to validate its effectiveness in integrating single-cell multimodal data. Additionally, to further evaluate scGCM’s performance in handling RNA and ADT mosaic data, we used different modalities for data from various batches and manually generated mosaic data for testing. We used the Cite-seq dataset of human peripheral blood mononuclear cells as the study subject.

In Figure S21a, we presented the performance metrics of various methods in data integration using bar charts, while in Figure S21b, we showed the UMAP visualizations of the latent representations learned by these methods. For the RNA and ADT paired data, we tested five different methods. The results showed that scGCM outperformed all other methods in ARI, with the most prominent performance. Although Mowgli and Snap’s performance lagged slightly, failing to reach the 0.7 level, Multigrate ranked first in NMI, with scGCM close behind, exceeding 0.8. While scGCM saw a slight decline in Graph Connectivity, with cLISI being fairly consistent across methods, scGCM performed best in iLISI, approaching 0.7. From the UMAP cell type plots generated from the experiments, scGCM effectively distinguished different cell types. In the UMAP plots for batch integration, scGCM performed excellently, effectively integrating data from different batches, whereas other methods failed to fully integrate batches 0 and 1.

### scCGM integration of trimodal paired data

**Fig. 3:**
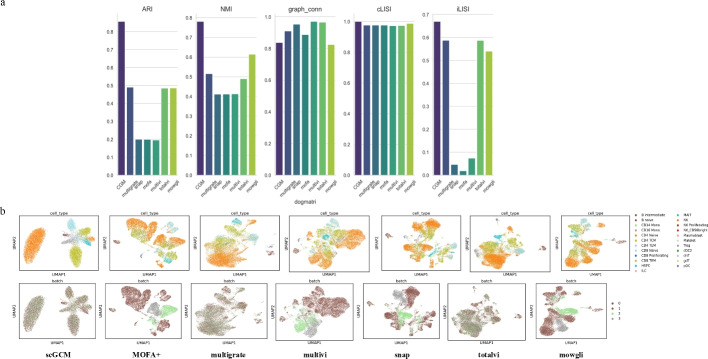
Evaluation obtained with scGCM on RNA,ATAC and ADT paired data. **a** benchmarking of performance in DOGMA-seq dataset. **b** UMAP visualization of cell embeddings obtained by scGCM and six other strategies in DOGMA-seq dataset

We benchmarked our model framework on two multimodal (RNA, ATAC, ADT) paired datasets to validate its performance in integrating single-cell multimodal paired data. Specifically, we used the DOGMA-seq and TEA-seq datasets of human peripheral blood mononuclear cells (PBMCs) for this study.

In Fig. 3a, we presented the performance metrics of various methods in data integration using bar charts, and in Fig. 3b, we showed the UMAP visualizations of the latent representations learned by these methods. When comparing the DOGMA-seq dataset with other methods, the results showed that scGCM’s ARI and NMI were significantly higher than other methods, both reaching around 0.8. In contrast, Multigrate, TotalVI, and Mowgli were in the second tier, with performances around 0.5, while Snap, MOFA+, and MultiVI had weaker results. Although scGCM’s performance in Graph Connectivity was slightly lower than other methods, all methods showed relatively consistent results for the cLISI metric. However, scGCM excelled in iLISI (Graph Integrated LISI), scoring above 0.6, with Multigrate, TotalVI, and Mowgli slightly below 0.6, while other methods were significantly lower. Additionally, scGCM performed exceptionally well in handling batch effects, especially in integrating batches 2 and 3, where scGCM achieved better integration, while MOFA+, MultiVI, Snap, and Mowgli performed poorly in this area. Multigrate, TotalVI, and Mowgli showed some improvement. For the performance evaluation and UMAP plots of the TEA-seq dataset, we presented the results in Figure S10–12. In this dataset, scGCM’s ARI and NMI both exceeded 0.8, significantly outperforming other methods, whose performance ranged between 0.6 and 0.7. However, it is worth noting that all methods performed relatively well in handling batch effects in the TEA-seq dataset. These results demonstrate that scGCM excels in integrating RNA, ATAC, and ADT multimodal paired data, effectively identifying cell types across various datasets and exhibiting stable integration performance. The outstanding performance of scGCM further validates its potential for application in multimodal single-cell data integration.

### scCGM integration of trimodal mosaic data

**Fig. 4:**
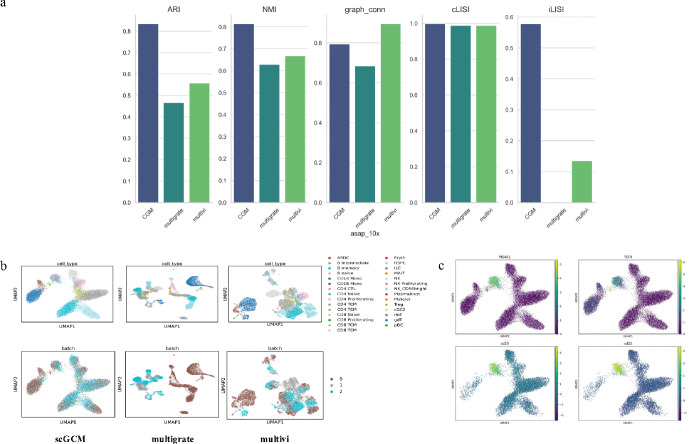
Evaluation obtained with scGCM on RNA, ATAC and ADT mosaic data. **a** benchmarking of performance in DOGMA-seq dataset. **b** UMAP visualization of cell embeddings obtained by scGCM and two other strategies in ASAP-10X dataset. **c** Gene expression of MS4A1 and TCF4 over all single-cell samples in ASAP-10X dataset. And protein weight of cd20 and cd22 in ASAP-10X dataset

We benchmarked scGCM on four sets of multimodal (RNA, ATAC, ADT) mosaic datasets to validate its effectiveness in integrating single-cell multimodal mosaic data. Specifically, we combined different batches of the DOGMA-seq and TEA-seq datasets of human peripheral blood mononuclear cells (PBMCs) to construct mosaic datasets. Additionally, we manually concatenated the ASAP-CITE dataset (RNA, ADT) of human peripheral blood mononuclear cells and the $10X\_PBMC$ dataset (RNA, ATAC), generating another set of mosaic datasets. And, we generated another set of mosaic datasets by concatenating the DOGMA-seq dataset (RNA, ATAC, ADT), Cite-seq dataset (RNA, ADT), and $10X\_PBMC$ dataset (RNA, ATAC). We compared MultiVI and Multigrate for the integration of tri-modal mosaic data. The remaining four methods were excluded from this evaluation because they are designed only on paired data and are incompatible with mosaic configurations.

In Fig. 4a, we presented the performance metrics of various methods in data integration, displayed in bar chart format; and in Fig. 4b, we showed the UMAP visualizations of the latent representations learned by these methods. We used two different model frameworks for comparative analysis. For the ASAP-10X dataset, scGCM’s ARI and NMI both exceeded 0.8, while the ARI of the other two methods was around 0.5, and their NMI did not exceed 0.7. In the iLISI metric, scGCM significantly outperformed other methods, reaching nearly 0.6, while in the cLISI metric, all methods performed similarly. Although MultiVI performed best in the Graph Connectivity metric, scGCM still reached around 0.8. The UMAP plots showed that scGCM performed well in clustering cell types and demonstrated an advantage in batch integration, whereas Multigrate and MultiVI failed to effectively integrate data from different batches. We generated gradient UMAP plots (Fig. 4c) using counts of marker genes and surface proteins. In these plots, it can be observed that MS4A1 remains a specific marker for B cells, while TCF4 is highly expressed in pDC cells, further highlighting the importance of these genes in immune cells. Notably, the CD20 protein encoded by MS4A1 is also highly expressed in B cells, and CD22 plays an important role in B cells as well. Thus, it can be concluded that by integrating data from different modalities, the similarities between them can be extracted, enhancing cell type identification. The complementary information between these data further improves the accuracy of distinguishing cell types.

## Conclusion and discussion

With the rapid development of sequencing technologies, multimodal data has become increasingly common, resulting in the accumulation of a large amount of multimodal mosaic data. However, there is a lack of analytical tools for integrating these data.

To analyze these multimodal mosaic data, we propose a modular framework based on a semi-supervised variational autoencoder (VAE). By using neighbor graphs and contrastive learning, we preserve the topological relationships between cells, eliminate batch effects, and enhance clustering precision, effectively facilitating downstream analyses.

In this study, we systematically evaluated the performance of the scGCM model in integrating multimodal single-cell data, particularly in handling RNA, ATAC, and ADT three-modal paired data and their mosaic datasets. Through benchmarking the three-modal mosaic dataset, we found that scGCM excelled in multiple key metrics, significantly outperforming the current state-of-the-art multimodal data integration methods. Additionally, scGCM also demonstrated excellent performance in UMAP clustering, allowing for clearer differentiation of cell types while effectively eliminating batch effects and successfully integrating data from multiple batches. Furthermore, UMAP visualizations of labeled genes and proteins indicate that the model maintains the specificity of features across modalities.

Despite scGCM’s outstanding performance on most metrics, there are still some areas that require further optimization. In particular, its performance in identifying less common cell types is slightly lower than that of some competing methods. Additionally, scGCM has not completely distinguished different cell types. We will continue to address these issues and aim to release an improved version of scGCM soon.

## Data Availability

No datasets were generated or analysed during the current study.
